# Is There an Association Between Migraine and Major Depressive Disorder? A Narrative Review

**DOI:** 10.7759/cureus.8551

**Published:** 2020-06-10

**Authors:** Saira Jahangir, Dennis Adjepong, Hieder A Al-Shami, Bilal Haider Malik

**Affiliations:** 1 Neuroscience, California Institute of Behavioral Neurosciences and Psychology, Fairfield, USA; 2 Neurological Surgery, California Institute of Behavioral Neurosciences and Psychology, Fairfield, USA; 3 Neurological Surgery, Al-Ahly Bank Hospital, Cairo, EGY; 4 Internal Medicine, California Institute of Behavioral Neurosciences and Psychology, Fairfield, USA

**Keywords:** major depressive disorder, genes, mental health, mental illness, mental disorder, migraine

## Abstract

Various studies on the association of migraine with depression are published. The comorbidity may upgrade health conditions up to a critical degree. Besides, the duration of symptoms and treatment may be prolonged. Moreover, these conditions can force substantial financial and social hardships on patients and their families. In this literature review, we intend to examine the evidence obtained on the possible associations between migraine and major depressive disorder (MDD). This review is focused on aminergic neurons. One of the variables associated with patients who experience both of these two diseases might have a history of assault. In migraine and MDD patients, genetic evidence, such as single nucleotide polymorphisms (SNP), was found to be one of the associations. Another theory concluded that actual headache diagnosed in patients who received no treatment manifests a history of anxiety, and later, the patients display severe somatic symptoms. In conclusion, there is a robust molecular genetic background, explaining the relationship between migraines and MDD. This correlated data renders a combination of both diagnoses as single separate entities. However, further studies are encouraged to point out the issue of treatment strategies.

## Introduction and background

"Depression affects almost 80% of migraine sufferers at one time or another" [1]. People with migraines, especially chronic migraines, are more likely to experience intense anxiety and suicidal tendencies [2]. In her book, Sarah Hackley disclosed her experience with lifelong migraines. She stated: "If we want to live a happy and joyful life with migraine, we must acknowledge and deal with the emotional realities of the disease [3]."

Migraine is one of the dominant causes of primary headache disorder worldwide [4]. Migraine is characterized by repetitive spells of headache episodes. Migraine is ranked eighth as one of the utmost excruciating diseases in females. Migraine is considered as the fourth most common pain syndrome, according to the 2012 Global Burden of Disease Study [5]. Migraine is a chronic condition that can affect patients' quality of life, functioning, and learned behavior [6].

Migraine manifests with various symptomatology degrees, which makes no stereotypical presentation and further leads to an overdose of prescribed medications [7]. Migraine headaches typically present along with nausea, vomiting, photophobia, and phonophobia [8].

A major depressive disorder (MDD) or depression is the leading cause of worldwide disability; comparatively, it exists side-by-side with academic deterioration, substance abuse, low self-esteem, a decrease in productivity, poor appetite, and sleep and self-destruction. Depression or major depressive disorder (MDD) presents along with deep emotions, loss of motivation, and contentment [9]. The prevalence of depression is approximately 20% more significant in females than in males [10].

Migraine with MDD presents as a mixed disease in individuals who experience one or more of these diseases [11]. Generally, patients with depression have a two-fold chance of developing migraines [12]. There are differences in the brain tissue volumes among patients who encounter these two diseases [13]. Also, there is a 40-50% heritable association associated with migraine and major depressive disorder [14]. Previous reports show that depression might accompany migraine episodes [15-16], while anxiety precedes the migraine [15-17].

By contrast, a study concluded that migraines could predict the onset of depression [18]. Frequently, there is a genetic interrelationship that describes that the same gene influence numerous conditions. In patients with migraines, depression is found to be a poor prognosis factor [19-20]. Amiri and coworkers published the famous metanalysis that links migraine and depression based on 16 selected articles [21]. They found that a pooled odds ratio (PR) of 1.95 and 95% confidence interval (CI) of 1.61-2.35 were obtained in studying the effects of migraine in depression. Friedman and coworkers [21] found a positive correlation between migraine and suicidal ideation. They reviewed over 14,000 patients and recorded odds of suicidal ideation to be 2.49-fold higher among individuals with migraine (OR: 2.49; 95% CI: 2.34-2.65) compared to those free of migraine.

In this traditional review, we intended to show some significant association between migraine and major depressive disorder based on their pathophysiology, biochemistry genetics, scientific analysis, clinical implication, cerebral cortex and thalamic changes, serotonin effect, pain as well as its prognosis on migraine and MDD.

## Review

Pathophysiology of disease

The knowledge of migraine pathophysiology in the present day is more understood now than a few years ago. The pain of anatomy and physiology for migraine structure suggests sub-cortical aminergic sensory arrangement, which broadly impacts the brain [22-23]. In the fundamentals of a migraine episode, the significant aspect is a headache and its associated symptomatology [24]. The data gathered displays that the core of migraine pathophysiology is thalamocortical dysrhythmia [25]. Traits of migraine as included in the International Classification of Headache Disorders - the second edition included episodes of headache that lasted from 4 to 72 hours and had the following features:

1. Migraine patients have many first-degree relatives who also suffer from this disease. 2. Functional magnetic resonance imaging (fMRI) measures the dysfunction of heat stimuli and pain modulation in the brain stem [26]. 3. Functional resonance imaging (fMRI) also detects the brain stem's excitable trigeminal vascular system [27].

On the other hand, there are several theories mainly focusing on the involvement of environmental factors associated with genetic and biochemical components for the pathophysiology of MDD [28]. In the serum of depressed patients, there is an increased level of inflammatory cytokines in the brain. These inflammatory biomarkers, which are interleukin-1 beta (IL-1B), interleukin-6 (IL-6), tumor necrotic factor-alpha (TNF alpha), high sensitivity C-reactive protein (hs-CRP), are found in depressed patients exclusively. The monoaminergic system remained as the primary treatment, which is evident from the treatment of MDD with an anti-inflammatory effect. This fact is still insufficient to adapt to a strict conclusion [28].

Pathobiology of disease

Single Nucleotide Polymorphism and Genome-Wide Association Study

In the past, research conducted for migraine and MDD measured a moderate heritability with an estimate of 30% to 50% between two diseases [28-31]. Data were collected from a previously published meta-analysis study for SNPs and gene-based analysis of genome-wide association study (GWAS) [32]. Besides, some genetic associations were observed between the patient who suffers from migraines and MDD. The overlap was found at two levels, the SNP and gene level. There were three novel independent genome-wide significant SNPs {rs146377178, rs672931, and rs11858956; located between cell division cycle associated 2 (CDCA2) Early B-cell Factor 2 (EBF2), within doublecortin domain containing 5 (DCDC5), and between ribosomal protein, large, P1 (RPLP1) and Transducin-like enhancer of split 3 (TLE3) respectively}. The two genome-wide significant genes are ankyrin repeat and death domain-containing 1A (ANKDD1B ) and potassium channel, subfamily K, member 5 (KCNK5). This study urged to determine the molecular overlap and to perform further combined analysis on the gene-based analysis of GWAS data. Finally, Yang et al. conducted a gene-based metanalysis [32]. There was a significant cross overlap between migraine and MDD at the gene and SNP level. There were three areas of considerable overlap. These were SNPs (rs146377178, rs672931, and rs11858956) between cell division cycle associated 2 (CDCA2) and early B-cell factor 2 (EBF2), within doublecortin domain-containing (DCDC5), and between ribosomal protein, large, P1 (RPLP1) and transducin-like enhancer of split 3 (TLE3), respectively, and two genome-wide significant genes ANKDD1B and KCNK5. The question about the categorization of migraine with MDD in a separate entity is real. Ligthart and colleagues, according to their genetic study, suggested an independent existence of migraine associated with MDD because their genetic background is 'purely' different from either type alone [33].

Serotonin Gene and Receptors

In the 1960s, researchers found that increase in 5-hydroxy indole acetic acid (5-HIAA) levels caused migraine attacks [34]. Diminished levels of serotonin in research subjects triggered severe depression [35]. Migraine and depression may be associated with a low level of 5-hydroxytryptamine (5-HT), or serotonin receptors. There is also evidence on the serotonin transporter gene alterations, the short allele is associated with risk of depression and also increases the occurrence of migraines [30]. Thus, from the latest evidence, it is indicated that both diseases do share common serotonin heritability factors. Panconesi and colleagues identified central and peripheral pathways for altered serotonin levels in migraineurs [36]. However, selective serotonin reuptake inhibitors (SSRIs) did not induce a headache as a side effect, nor were they part of the treatment. How can this be explained? These drugs, as well as serotonin agonists, can increase transmitter concentrations extracellularly but taking into consideration the following differences: (1) SSRIs tend to produce a small increase in the extracellular neurotransmitter, whereas releasers tend to produce robust gains (2) Fenfluramine, nor-fenfluramine, meta-Chlorophenylpiperazine (mCPP) and 3,4-Methyl​enedioxy​methamphetamine (MDMA) are potent 5-HT releasers via carried-mediated exchange mechanism involving serotonin transporter (SERT) sites in the brain, (3) Fenfluramine and meta-Chlorophenylpiperazine (mCPP) also have direct agonist actions at 5-hydroxytryptamine (5-HT) receptors. Fenfluramine, especially their metabolite nor-fenfluramine, are potent serotonin receptors (5-HT2C and 5-HT2B receptor agonists). Meta-chlorophenylpiperazine (mCPP) is a very potent agonist in human serotonin receptor (5-HT2C and 5-HT1A) [36].

*Morphological Changes in Cerebral Cortex and Thalamus* 

Cerebral cortex micro-architecture is different in patients with migraines and MDD compared to the patients who are suffering from a single disease [37-38]. In patients with both ailments, we also notice a discordant developmental track of the fusiform gyrus and detectable changes in the thalamus [37]. Changes are also evident in the control of pain and mood perceived by these comorbid individuals. The resting-state functional magnetic resonance imaging provides evidence that affection was mainly in the left medial prefrontal cortex (m PFC) [37]. During this study, the medial prefrontal cortex was active at rest, but during the time of cognitive and emotional activity, it became inhibited. The intrinsic brain activity of the left medial prefrontal cortex was increased in migraineurs as well. The m PFC is the anterior node of the default network mode (DNM), which has a low-frequency oscillation. The relationship between cerebral cortex thickness and cerebral activity needs further exploration to understand the co-pathogenic relationship between patients' cerebral function who was having depression with migraines [37-38]. The comorbid condition of depressive and migraine patients exhibits a marked decrease in the intrinsic brain activity in the thalamus [39]. Another study determined that some older people who do not have dementia but have a migraine and MDD had an overall reduced total brain volume as well as gray and white matter compared to patients who presented with either of the diseases [40].

Treatment

Tricyclic antidepressant is one of the anti-inflammatory medicines [41]. It can be effective for depression and migraines [41]. One of the Tricyclic Antidepressants commonly use is amitriptyline [42]. The mechanism of action of Amitriptyline ​​​​​, a tertiary tricyclic antidepressant (TCA), is to inhibit Serotonin (5HT) and Nor-epinephrine (NE) reuptake. Amitriptyline provides the best evidence in clinical practice [41]. If one cannot tolerate amitriptyline, then nortriptyline is an alternative [42]. Besides tricyclic antidepressants, Serotonin Non-Reuptake Inhibitor (SNRI) has strong evidence for efficacy in a patient with comorbid depression and migraine [42]. There is a further need to research on the tolerability and efficacy of Serotonin Non-Reuptake Inhibitor (SNRI) to prevent migraine headaches [42].

Further research

Although we have found multiple factors associated with migraine and MDD, this review illustrates the need to find the molecular and genetic overlap between these two diseases. We suggest performing further combined analysis of the GWAS on patients with migraine and MDD. Also, to find one anti-inflammation treatment for depression and cortical sensory aminergic association with migraine to achieve a treatment plan for patients who experience both migraine and MDD.

In this review, we reflected in proposing several equipotential details of evidence connecting migraine and MDD. We highlighted that aminergic neurons were one of the variables when we reviewed pathophysiology. We observed genetic prediction in migraine and MDD patients, such as SNPs, as one of the associations detected. The scientific analysis considered and analyzed active headache patients who had no treatment and suffered from anxiety and severe somatic symptoms. In the brain of migraine and MDD patients, we recognized alterations in the prefrontal cortex, particularly the left medial prefrontal cortex. We have additionally seen transformations in the temporal-occipital gyrus and thalamus of patients who had a migraine and MDD. There is a diminished level of serotonin observed in patients who are comorbid with migraine and MDD. In this paper, we suggest discussing correlated determinants discovered among patients diagnosed with both of the diseases. In the future, this review might support clinicians in recognizing while screening patients with either migraine or MDD to implement more trustworthy management; consequently, these patients would be able to achieve liver their day to day life abundantly.

## Conclusions

There is a robust molecular genetic background explaining the relationship between migraine and MDD. This correlated data renders a combination of both diagnoses as a single separate entity. However, further studies are encouraged to point out the issue of treatment strategies.
